# Conferring of drought tolerance in wheat (*Triticum aestivum* L.) genotypes using seedling indices

**DOI:** 10.3389/fpls.2022.961049

**Published:** 2022-07-22

**Authors:** Hafiz Ghulam Muhu-Din Ahmed, Yawen Zeng, Adnan Noor Shah, Muhammad Majid Yar, Aziz Ullah, Muhammad Ali

**Affiliations:** ^1^Department of Plant Breeding and Genetics, Faculty of Agriculture and Environment, The Islamia University of Bahawalpur, Bahawalpur, Pakistan; ^2^Biotechnology and Germplasm Resources Institute, Yunnan Academy of Agricultural Sciences, Kunming, China; ^3^Department of Agricultural Engineering, Khwaja Fareed University of Engineering and Information Technology, Rahim Yar Khan, Pakistan; ^4^Department of Plant Breeding and Genetics, Faculty of Agriculture, University of Sargodha, Sargodha, Pakistan; ^5^Department of Environmental Sciences, Faculty of Agriculture and Environment, The Islamia University of Bahawalpur, Bahawalpur, Pakistan

**Keywords:** wheat, drought stress, seedling stage, multivariate analysis, correlation

## Abstract

Wheat is the most widely grown and consumed crop because of its economic and social benefits. This crop is more important globally for food and feed, and its productivity is particularly vulnerable to abiotic factors. In this study, 40 wheat genotypes were studied to access the drought tolerance level using completely randomized design (CRD) in 250 ml disposable cups through morpho–physiological attributes at seedling stage. The wheat germplasm was tested under normal and two drought stress level D1 (50% field capacity) and D2 (75% field capacity) for different seedling attributes such as germination percentage (GP), chlorophyll content (CC), shoot length (SL), root length (RL), shoot fresh weight (SFW), root fresh weight (RFW), seedling fresh weight (SDFW), shoot dry weight (SDW), root dry weight (RDW), relative water content (RWC), root/shoot ratio (RS), and seedling dry weight (SeDW). The results of analysis of variance (ANOVA) and spider analysis indicate that significant amount of genetic variation was present and behavior of studied germplasm showed different behavior in different environment. The correlation analysis showed that root length has significantly positive association with root/shoot ratio, dry weight, and fresh weight while negatively correlated with shoot length and relative water content. Based on the positively associated traits, the studied genetic material would improve genetic gain for drought tolerance. The multivariate analysis showed that out 13 principal components only five PCs were significant and has eigenvalue > 1, cumulatively showed 82.33, 83.07, and 97.34% of total variation under normal, D1 and D2 conditions, respectively. Significantly, the result of spider graph and multivariate analysis showed that genotypes G47, G48, G65, G68, and G80 performed well in all drought stress conditions and considered as drought-tolerant genotypes. The best performing genotypes can be used in future breeding programs. The selection on the bases of studied attributes is effective for development of drought-tolerant and high-yielding varieties for sustainable food security.

## Introduction

Wheat (*Triticum aestivum* L.), originating from South Western Asia, is cultivated throughout the world. It is known as the king of cereals and consumed as a staple crop by one-third of the global population. According to an estimate, wheat is the second most commonly grown crop in the world (Ahmed et al., 2022a). Bread wheat provides approximately 70% of calories and 80% of protein (Asl et al., 2011). Wheat grain contains 8–17% protein, 60–80% carbohydrates (mostly starch), 1.5–2% minerals, 1.5–2% lipids, 2.2% crude fibers, vitamins (B-complex and E), and almost all essential amino acids (Shewry and Hey, 2015). Gluten is a protein complex found in bread wheat that accounts for 75–85% of the total protein (Shewry, 2019). The wheat grain protein has different chemical and physical properties. To achieve the optimum genotypes on this aspect, it is vital to understand the inheritance mechanism of wheat grain and quality attributes (Hussain et al., 2012).

To fulfill the needs of the world's fast-increasing population, wheat production must double by 2050 (FAO, 2021). According to the FAO December Quarterly Report for 2021 wheat is cultivated on 223.36 Mha and produced 777.9 Mt of wheat worldwide. Over the previous year's planted area of 8,805 thousand hectares, the area under cultivation improved by 4.2% to 9,178 thousand hectares in 2020–2021. In Pakistan, wheat production reached an all-time high of 27.293 Mt worldwide (FAO, 2021). In Pakistan, the wheat crop was cultivated on 3,335 thousand hectares with a 9.9% increase in area from the previous year and shows a 13.6% increase in production which is 8.419 Mt from the previous year 7.414 Mt production (GOP, 2021).

Pakistan has all the necessary conditions for better yields, such as soil, water, and environment, yet the output is lower than other large wheat-producing countries. Wheat production can only be enhanced by increasing productivity rather than increasing acreage. As a result, increasing wheat output is critical to ensuring long-term food security (Ahmed et al., 2022a). Wheat crop stability is critical among cereal crops due to its high consumption and nutritional value. Wheat scientists face new problems in breeding wheat varieties with a higher yield, quality, and resistance to biotic and abiotic pressures as a result of the rapid growth in population and improved lifestyle (Uzair et al., 2016). Poor-quality seed, non-recommended seeding methods, late sowing, non-recommended sowing methods, poor soil monitoring, unbalanced fertilizer application, inappropriate weed eradication, diseases, heat, water scarcity, and drought stress climate change, and all these factors are extreme that lead to low yield. (Abbas et al., 2005; Bashir et al., 2006). Due to the continuous increase in the population of Pakistan, it is the need for time to improve the yield of staple food.

Among different abiotic stresses, drought stress has harmful effects on growth and development of wheat crop. Drought tolerance is a highly complex trait and one of the important components of yield stability in wheat (Ahmed et al., 2022a,b). Drought stress negatively affects the physio-morphological attributes like; shoot length, root length, relative water content, chlorophyll content, leaf area in wheat crop. It is one of the most common causes of crop loss around the world, lowering average yield by more than 50% for agricultural plants (Wang et al., 2003; Bayoumi et al., 2008). Drought can damage a plant at any point in its life cycle, but specific stages, such as seedlings, are critical (Fang and Xiong, 2015).

Drought is the biggest issue that affect agricultural production all over the world. Drought or water deficit is edaphic stress that affects plant growth and dramatically reduce the agricultural productivity in many parts of the world (Comas et al., 2013). This stress can simultaneously affect many traits through agronomic, morphological, physiological, biochemical, and metabolic changes which occur in all plant tissues and ultimately reduce yield performance (Cochard et al., 2002). Water shortage conditions reduce yield; hence, it is estimated that water shortages are responsible for 17–70% of production losses. However, the water shortages in developing countries have reduced wheat yields from 50 to 90% of their irrigated potential (Cochard et al., 2002). Drought stress causes damaged germination and poor growth. The researcher Fathi et al. (2016) also found that the drought stress causes a significant reduction in germination and seedling growth due to a lack of water. Wheat is very sensitive to drought during the early seedling stages and germination, unlike many other crops. Seedlings of crops plant are extremely sensitive to drought stress conditions (Mahpara et al., 2022). Drought reduces water intake and lowers seedling vigor, both of which are harmful to germination (Datta et al., 2011). Drought stress affects water balance, disrupts metabolic reactions at the cellular level, reduces ATP synthesis and respiration, and results in poor seed germination (Upadhyaya et al., 2021).

Wheat genotypes were screened for drought stress tolerance and gives higher production. The current study focus on screening of wheat genotypes on morpho–physiological attributes such as germination percentage (GP), chlorophyll content (CC), shoot length (SL), root length (RL), shoot fresh weight (SFW), root fresh weight (RFW), seedling weight (SDW), shoot dry weight (SDW), root dry weight (RDW), *R* = relative water content (RWC), root/shoot ratio (RSR), and Seedling dry weight (SeDW) against drought stress conditions. The main objectives of this study were listed as follows: (i) Screening of drought-tolerant wheat cultivars ii); Identification of drought susceptible cultivars; (ii) Assessment of seedling attribute against drought stress. Wheat breeder should use these findings to select are screen drought-tolerant cultivars and develop high yielding cultivars in future.

## Material and methods

The research work was carried out at the Research area of Department of Plant Breeding and Genetics (PBG), The Islamia University of Bahawalpur, Pakistan (29.24°N, 71.41°E) during November 2021. In this study, 40 wheat genotypes were grown using complete randomized (CR) design under normal and drought stress conditions to evaluate the wheat genotypes on morphological and physiological indices against drought stress at seedling stage. These genotypes were taken from Regional Agriculture Research Institute (RARI) Bahawalpur, Pakistan. The name, pedigree (if available), and origin of selected wheat genotypes are listed in Supplementary Table S1. Each genotype was grown in 250-ml disposable water cups containing sand mixture developed and described previously by the researcher Fan et al. (2015). Five seeds were sown in each cup, and after germination, thinning was done to one plant. After sowing, the genotypes are subjected to three treatments, with one set of genotypes regularly watered (100% of field capacity), the other two sets were kept in water deficit conditions (50 and 75% of field capacity), which was denoted by N, D1, and D2, respectively. A pressure chamber apparatus was utilized to measure the field capacity (FC) of the soil used in this study (Gugino et al., 2009; Moebius-Clune, 2016). Before the application of stressed condition, 20-ml Hoagland (Hoagland and Arnon, 1950) solution was applied to all treatment to boast the germination growth of seedling. The first dose of water was applied 15 days after the date of sowing. A total of three doses (40 ml/dose) were applied at 5-day intervals for each cup. Upon germination, the data of germination percentage were recorded and thinning was performed on one plant in each cup. After 30 days, the wheat plants had 3–4 leaves and the data were recorded. A ruler was used to measure the root length (RL) and shoot length (SL) (in cm). The electric weight balance was used for the measurement of shoot fresh weight (SFW), root fresh weight (RFW), seedling fresh weight (SDFW), seedling dry weight (SeDW), shoot dry weight (SDW), and root dry weight (RDW). These traits measured in grams. The root/shoot ratio was calculated by dividing root length to shoot length. The Hansatech SPAD meter model CL-01 was used for chlorophyll content measurement. The germination percentage was recorded and calculated using the following equation (Ellis and Roberts, 1981; Ruan et al., 2002).


$$\begin{array}{l} \text{Germination percentage }\left(\text{GP}\right)=\frac{\text{Number of germinated seed }}{\text{Total Number of seed tested}}\;\times \;100 \end{array}$$

The relative water content was measured by using the formula (Ahmed et al., 2019).


$$\begin{array}{l} \text{RWC}=\frac{\left(\text{Fresh weight Dry weight}\right)\;}{\left(\text{Turgid weight Dry weight}\right)\;}\times \;100 \end{array}$$

The collected data was analyzed through analysis of variance (ANOVA) technique (Steel and Torrie, 1980) using Statistix 10.0 software to check significant difference in studied genotypes. The significance level, α = 0.01, was used for the highly significant effect while α = 0.05 was used for the significant effect among genotypes. To study the association between the significant character and the genotypes, the correlation analysis was performed through Pearson correlation formula.


$$\begin{array}{l} {\text{r}}_{\text{xy}}=\frac{\sum ({\text{x}}_{\text{i}}-\bar{\text{x}})({\text{y}}_{\text{i}}-\bar{\text{y}})}{\sqrt{\sum {\left({\text{x}}_{\text{i}}-\bar{\text{x}}\right)}^{2}}\sum {\left({\text{y}}_{\text{i}}-\bar{\text{y}}\right)}^{2}} \end{array}$$

Here, *x* is the first variable and *y* is the second variable.

The data of studied traits represented through spider graphs analysis (Ahmed et al., 2020). To construct spider graphs, XLStat 2014 was used. The recorded data was summarized through multivariate analysis (Ahmed et al., 2019) using software Minitab 16. The significance level α = 0.01 is used for highly significant effect while α = 0.05 for significant effect among genotypes. The principal components (PCs) have eigenvalue more than 1 is considered as significant. Based on the results of ANOVA, PCA, and correlation analyses, the wheat genotypes were screened as drought-tolerant and susceptible cultivars.

## Results

Analysis of variance (ANOVA) result for all examined traits is presented in Table 1, which shows highly significant difference among the traits and genotypes under normal and drought conditions. The mean value of different traits such as germination percentage, shoot length, shoot fresh weight, shoot dry weight, chlorophyll content, and root/shoot ratio showed decreasing trends under drought conditions in this study (Supplementary Table S2). The spider graph also showed a significant difference among the genotypes under stress conditions. It represents the mean values to a central point for the studied traits.

**Table 1:** Analysis of variance (ANOVA) of all studied traits under normal and stress conditions.

**SOV**	**DF**	**GP**	**SL**	**RL**	**SFW**	**RFW**	**SDW**	**RDW**	**CC**	**R/SR**	**SDFW**	**SeDW**	**RWC**
Treatment	2	3332.53^**^	799.97^**^	318.24^**^	1.1809^**^	0.04973^**^	0.00798^**^	0.06463^**^	53.403^**^	17.213^**^	0.7825^**^	0.11407^**^	3223.47^**^
Genotype	39	153.47^**^	10.58^**^	9.53^*^	0.0043	0.00120^**^	0.00027^**^	0.00036^**^	0.272^**^	0.059	0.0072^*^	0.00100^**^	327.62
G*T	78	31.31	8.95^**^	8.04	0.0031	0.00072	0.00011	0.00032^**^	0.202^**^	0.08	0.0043	0.00044	333.35
Error	240	25.76	6	6.41	0.0042	0.0006	0.0001	0.00019	0.034	0.195	0.0045	0.00038	385.55
Total	359												

SOV, source of variation; DF, degree of freedom; GP, germination percentage; SL, shoot length; RL, root length; SFW, shoot fresh weight; RFW, root fresh weight; SDW, shoot dry weight; RDW, root dry weight; CC, chlorophyll content; SC, stomatal conductance; VI= vigor index; GI, germination index; RWC, relative water content;

** Highly significant (0.01);

* significant (0.05).

### Performance of studied genotypes

**Figure 1:**
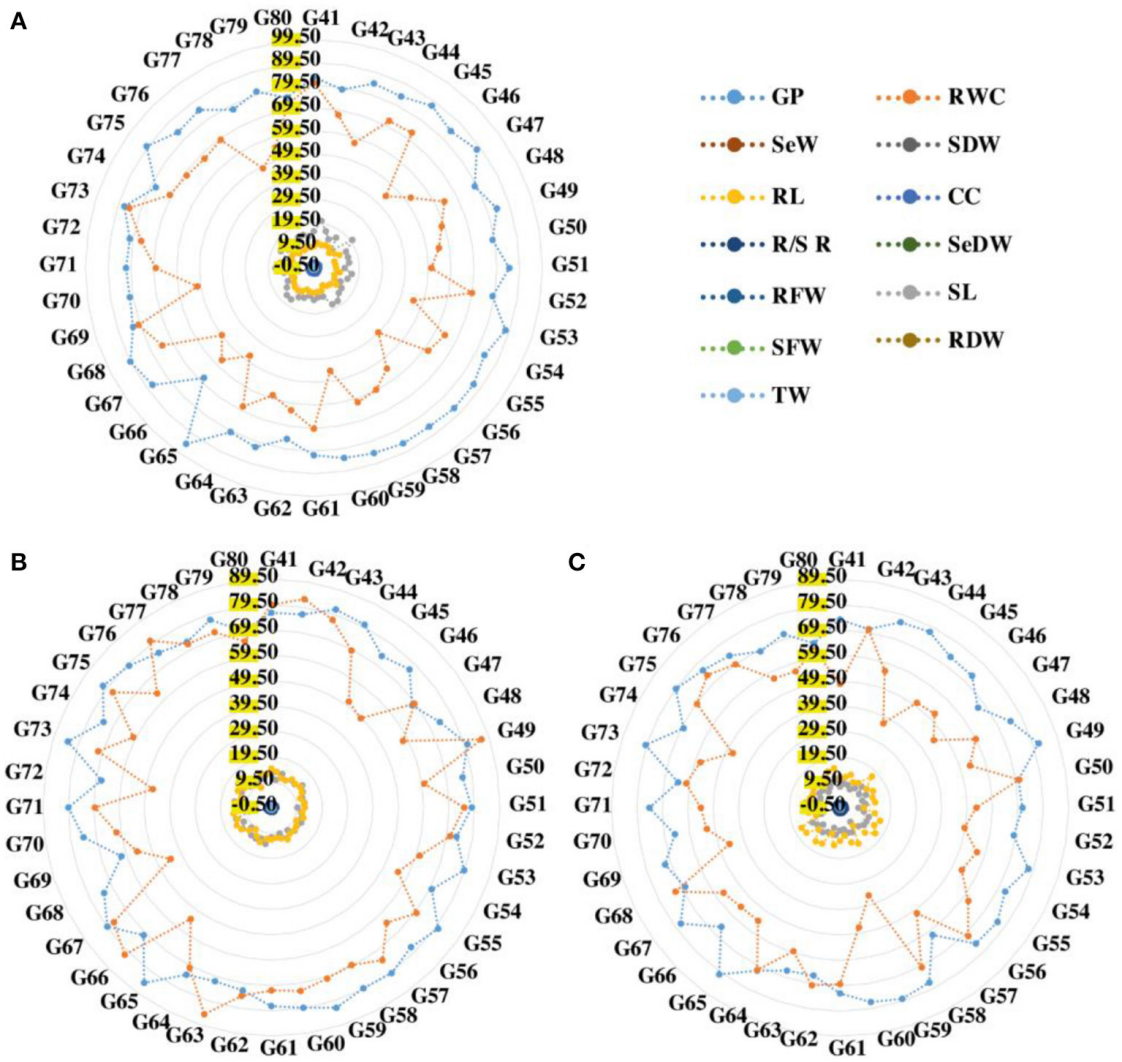
Radar analysis of 40 wheat genotypes under normal **(A)**, 50% drought **(B)**, and 75% drought **(C)** conditions. GP, germination percentage; CC, chlorophyll content; SL, shoot length; RL, root length; SFW, shoot fresh weight; RFW, root fresh weight; SDFW, seedling fresh weight; SDW, shoot dry weight; RDW, root dry weight; RWC, relative water content; R/S R, root shoot ratio; SeDW, seedling dry weight.

**Figure 2:**
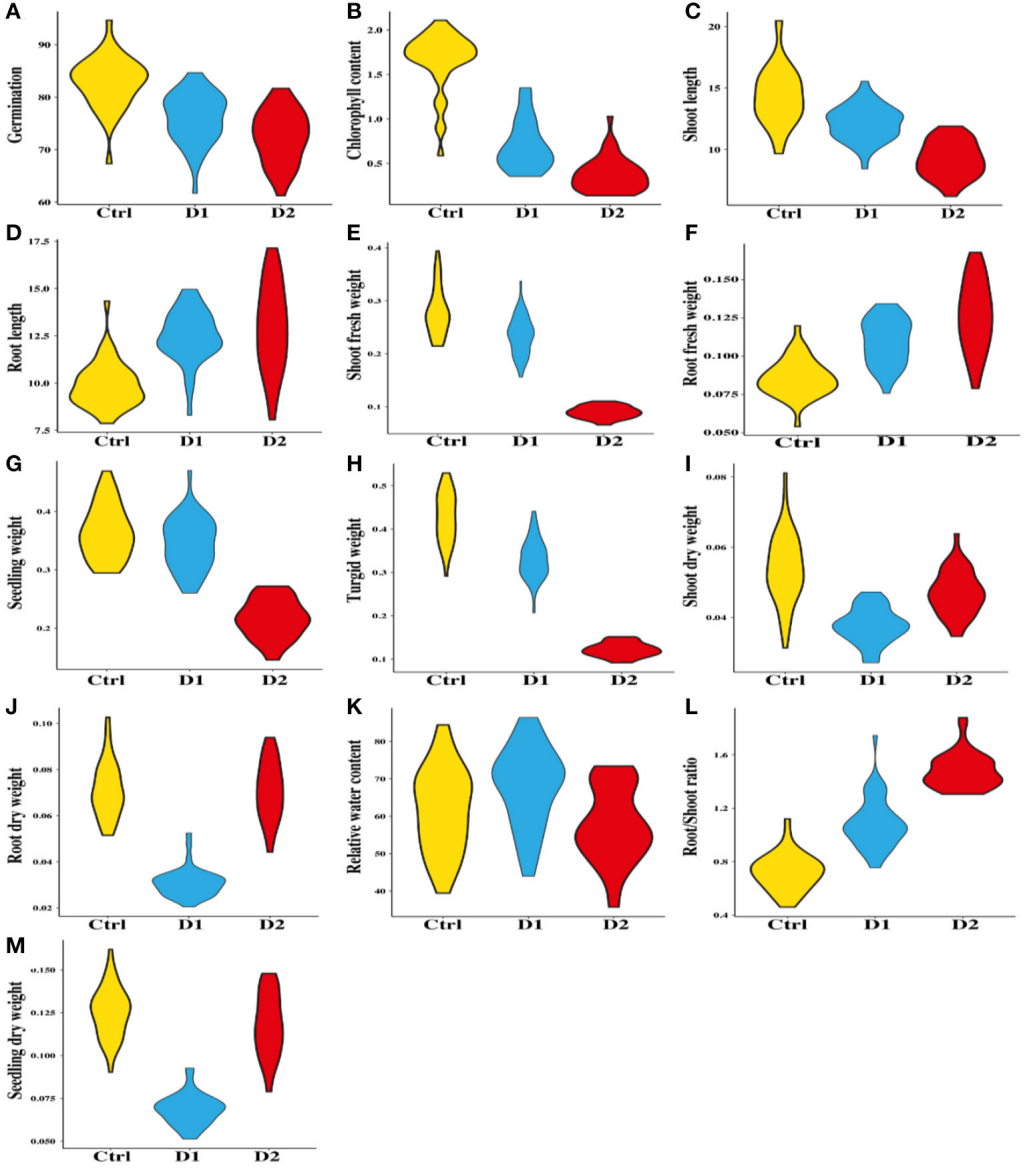
Comparison of morpho–physiological traits among 40 genotypes of wheat under control, D1, and D2 stressed conditions.

The data for the germination percentage were recorded under both normal and drought stressed conditions. In normal conditions, the germination percentage of wheat seedlings ranged67.33–94.67% with a mean value of 82.86%, according to the data shown in Supplementary Table S2. It ranged 61.67–84.67 in drought conditions (D1) with a mean germination percentage of 76.02%. In drought conditions (D2), the germination percentage ranged 61.22–81.67% with a mean germination percentage of 72.50%. In the germination percentage, genotype G65 showed the maximum value and genotype G66 showed a low value under normal condition (Figure 1). In drought condition (D1), genotype G65 showed the maximum value of germination percentage and genotype G69 showed the minimum value of germination percentage. In drought condition (D2), the result of germination percentage showed genotype G49, followed by G73 which had showed higher values, and genotype G57 followed by G72 showed lower values of germination percentage (Figure 2; Table 2)

**Table 2:** Performance of wheat genotypes under normal and different levels of drought stress.

**Trait**		**Best performance**	**Worst performance**
**GP**	N	G65 (94.67) followed by G75 (90.00)	G66 (67.33) followed by G62 (75.33)
	D1	G65 (84.73) followed by G65 (83.67)	G69 (61.67) followed by G72 (67.33)
	D2	G49 (81.67) followed by G73 (80.00)	G57 (61.22) followed by G72 (64.00)
**CC**	N	G58 (2.11) followed by G48 (2.03)	G43 (0.59) followed by G69 (0.89)
	D1	G48 (1.35) followed by G42 (1.31)	G63 (0.35) followed by G68 (0.36)
	D2	G60 (1.02) followed by G48 (0.75)	G63 (0.13) followed by G78 (0.14)
**SL**	N	G42 (20.47) followed by G47 (20.33)	G69 (9.67) followed by G46 (10.13)
	D1	G75 (15.6) followed by G47 (14.5)	G70 (8.4) followed by G51 (9.8)
	D2	G68 (11.9) followed by G46 (11.4)	G57 (6.2) followed by G51 (6.8)
**RL**	N	G48 (11.97) followed by G55 (11.90)	G58 (7.87) followed by G78 (8.27)
	D1	G41 (15.0) followed by G68 (14.7)	G78 (8.3) followed by G71 (9.7)
	D2	G46 (17.2) followed by G68 (16.7)	G57 (8.1) followed by G51 (8.8)
**SFW**	N	G71 (0.394) followed by G80 (0.371)	G53 (0.215) followed by G47 (0.217)
	D1	G79 (0.337) followed by G80 (0.290)	G57 (0.156) followed by G68 (0.179)
	D2	G53 (0.107) followed by G67 (0.101)	G51 (0.066) followed to G57 (0.067)
**RFW**	N	G72 (0.120) followed by G48 (0.108)	G58 (0.054) followed by G78 (0.069)
	D1	G48 (0.134) followed by G79 (0.133)	G71 (0.076) followed by G63 (0.086)
	D2	G46 (0.168) followed by G68 (0.163)	G51 (0.086) followed by G57 (0.079)
**SDFW**	N	G71 (0.469) followed by G75 (0.449)	G53 (0.295) followed by G58 (0.298)
	D1	G79 (0.470) followed by G76 (0.404)	G57 (0.260) followed by G71 (0.272)
	D2	G46 (0.272) followed by G68 (0.268)	G57 (0.146) followed by G51 (0.152)
**SDW**	N	G42 (0.081) followed by G67 (0.072)	G69 (0.031) followed by G57 (0.037)
	D1	G55 (0.047) followed by G72 (0.046)	G77 (0.0269) followed by G57 (0.027)
	D2	G59 (0.064) followed by G80 (0.057)	G51 (0.035) followed by G57 (0.0356)
**RDW**	N	G57 (0.103) followed by G56 (0.098)	G67 (0.052) followed by G73 (0.053)
	D1	G59 (0.052) follower by G54 (0.048)	G71 (0.020) followed by G63 (0.023)
	D2	G46 (0.094) followed by G68 (0.091)	G57(0.044) followed by G51(0.048)
**RWC**	N	G73 (84.44) followed by G41 (80.56)	G56(39.50) followed by G46(44.00)
	D1	G49 (86.38) followed by G63 (85.04)	G68 (43.96) followed by G72 (46.84)
	D2	G76 (73.42) followed by G68 (72.31)	G59 (35.71) followed by G44 (36.89)
**RSR**	N	G72 (1.12) followed by G69 (1.00)	G58 (0.46) followed by G47 (0.50)
	D1	G70 (1.75) followed by G51 (1.40)	G78 (0.75) followed by G65 (0.79)
	D2	G52 (1.88) followed by G47 (1.83)	G42 (1.30) followed by G51 (1.31)
**SeDW**	N	G56 (0.162) followed by G42 (0.151)	G69 (0.090) followed by G73 (0.102)
	D1	G54 (0.093) followed by G59 (0.092)	G77 (0.051) followed by G71 (0.052)
	D2	G46 (0.148) followed by G68 (0.146)	G57 (0.079) followed by G51 (0.084)

GP, germination percentage; CC, chlorophyll content; SL, shoot length; RL, root length; SFW, shoot fresh weight; RFW, root fresh weight; SDFW, seedling fresh weight; SDW, shoot dry weight; RDW, root dry weight; RWC, relative water content; R/S R, root shoot ratio; SeDW, seedling dry weight.

The chlorophyll content of wheat under normal conditions ranged 0.58–2.11 with a mean value of 1.66. In D1 conditions, it ranged 0.35–1.35 with a mean value of 0.71. The trait varied from 0.13 to 1.02 in D2 conditions, with a mean value 0.37. In normal conditions, genotype G58 exhibited the high value. However, genotype G43 exhibited the low value. Genotype G73 had the highest value for chlorophyll contents and G63 had the lowest value for Chl contents in drought condition (D1) (Figure 1). In D2 drought condition, the Chl showed genotype G60 followed by genotype G48, which showed high value, and genotype G63 followed by G78 showed low values.

Shoot length is an important trait that was affected by drought stress. Shoot length increased from 9.67 to 20.47 cm and showed mean value of 14.36 in normal conditions and from 8.40 to 15 60 cm with mean value of 12.15 in D1 conditions while in D2 the value increased from 6.2 cm to 11.90 cm and mean value (9.21) as listed in Supplementary Table S2. Genotypes G42 and G75 had maximum shoot lengths of 20.47 and 15.60 cm, respectively, under normal and drought conditions (D1) while G69 and G70 had the low value of shoot length of 9.67 and 9.67 cm, respectively, in normal and drought stress (D1). Genotype G68 (11.9 cm) had the high mean value and G57 (6.20 cm) had the low mean value. The root length of the given data ranged 7.87–14.33 cm with mean value of 9.92 in normal conditions and ranged 8.30–15.00 cm with mean value of 12.49 in D1 conditions; and in D2, the value ranged 8.10–17.20 cm and had the mean value 12.94. Both genotypes G48 and G41 had the maximum root lengths of 14.33 and 15.0 cm, respectively, under normal and drought conditions (D1) while genotypes G58 and G78 had the shortest root lengths of 7.87 and 8.30 cm, respectively, under normal and drought conditions (D1). Genotype G46 had the higher mean value of 17.20 cm and genotype G57 had the low mean value of 8.10 cm. In this trait, genotype G46 showed the best root length of 17.20 cm.

For normal, D1, and D2 levels, the data analysis showed significant differences in fresh root weight (Figure 2). The shoot fresh weight varied from 0.215 g to 0.394 g in normal conditions, with mean value of 0.279 g; and ranged 0.156–0.3370 g under drought conditions (D1), with mean value of 0.234 g, while ranged 0.066–0.111g in D2 conditions, with mean value of 0.089 g (Figure 1). In normal and D1 conditions, genotypes G71 and G79 had the maximum shoot fresh weight with a range of 0.394–0.337 g, respectively, while genotypes G53 and G57 had the minimum value for fresh weight that range 0.215–0.066 g. In D2 conditions, genotype G46 had high shoot fresh weight value of 0.272 g and genotype G57 had low mean value of 0.066 g.

According to the data listed in Supplementary Table S2 the root fresh weight (RFW) of wheat under normal conditions ranged 0.054–0.120 g with a mean value of 0.085 g. In D1 conditions, it ranged 0.0760–0.134 g with a mean of 0.109 g. The RFW ranged 0.079–0.168 g in D2 conditions with a mean of 0.126 g. In this trait, genotype G72 has the highest performance, while genotype G58 has the lowest performance under normal circumstances. The G48 had the highest RFW and G71 had the lowest RFW in D1 condition. Under D2 condition, genotype G46 had maximum mean value (0.168 g) and Genotype G51 had lower mean performance (0.086 g) as listed in Table 2. In this experiment, the root fresh weight increased under the drought stress. Among all the genotypes, genotype G72 had the highest RFW; however, genotype G58 showed the lowest RFW under normal condition (Figure 1).

The seedling fresh weight under normal conditions ranged 0.295–0.469 g with the mean value of 0.365 g. Under D1 conditions, the value ranged 0.260–0.470 g with the mean value of 0.344 g. In D2 conditions, the value ranged 0.146–0.272 g with the mean value of 0.216 g. Genotype G71 had high value (0.469) and genotype G53 had low value (0.295) under normal conditions, while genotype G79 had a high value (0.470) and genotype G57 had a low value 0.260 under drought conditions (D1). In D2 condition, genotype G46 exhibited high performance (0.272) and genotype G57 exhibited low value (0.146) for mean performance.

Shoot dry weight differed significantly under normal and drought stress levels as the results showed by the analysis of variance. Shoot dry weight is also an important trait that is affected by drought stress. As described in Supplementary Table S2, dry root weight in all varied in all conditions. Its value ranged 0.031–0.081 g in normal conditions with a mean value (0.054 g) and ranged 0.027–0.047 g in drought (D1) stress conditions with a mean of 0.037 g. In D2 stress conditions, the value ranged 0.035–0.064 g with mean value 0.046 g. Genotypes G42 and G55 had the highest dry shoot weight, 0.081 and 0.047, respectively, under normal and drought stress (D1) conditions while genotypes G69 and G77 had the lowest dry shoot weight, 0.031 and 0.0269, under normal and drought stress conditions (D1). Genotype G59 had the high mean value (0.064) and genotype G51 indicated the lower mean value (0.035) under D2 stress condition.

The analysis of data showed that changes in dry root weight between the normal and stress levels were very significant. The root dry weight (RDW) of wheat under normal conditions ranged 0.052–0.103 g with a mean value of 0.071 g as shown in Supplementary Table S2. Under D1 condition, the value ranged 0.020–0.052 g with a mean value of 0.031 g. In D2 drought conditions, RDW ranged 0.044–0.094 g with a mean value of 0.071 g. Under normal circumstances, genotype G57 had the highest result (0.103) but genotype G67 had the lowest result (0.052) among the studied data. In D1 drought condition, genotype G59 (0.052) had the highest value for RDW and G71 (0.020) had the lowest for RDW. The root dry weight showed genotype G46 performed best (0.094) and genotype G57 performed worst (0.044) in D2 drought condition.

Supplementary Table S2 clearly showed that in normal conditions, the value of RWC varied from 39.50 to 84.44 with a mean value of 61.53 and genotype G73 had the maximum value (84.44) and genotype G56 had minimum value (39.50) of RWC. In D1 drought level, the RWC ranged 43.96–86.38 with a mean value of 67.87 and genotype G49 had the maximum mean value of 86.38 and genotype G68 had the minimum mean value 43.96 for RWC. The RWC in D2 drought level, the value ranged 35.71–73.42 with a mean value of 57.57 (Figure 1). The maximum RWC value was found in genotype G76 (73.42). Whereas the minimum RWC value was found in genotype G59 (35.71). The Supplementary Table S2 showed that the root/shoot ratio ranged 0.460–1.120 in the normal conditions with mean value (0.723) and the value ranged 0.750–1.750 in drought D1 conditions with the mean value of 1.089. In D2 conditions, it ranged 1.30–1.88 with a mean value of 1.48. Under drought conditions (D1), genotype G70 had the highest root/shoot ratio (1.75) while genotype G78 had the lowest root/shoot ratio (0.75). Genotype G52 showed high value (1.88) and genotype G42 had low value (1.30) for root/shoot ratio.

The value of SeDW in the studied genotypes that was tested ranged 0.090–0.162 in normal conditions with a mean value of 0.125 and it ranged 0.051–0.093 under D1 conditions with a mean value of 0.068 as shown in Supplementary Table S2. The values in D2 conditions, on the other hand, varied from 0.079 to 0.148 with a mean value of 0.117 (Figure 2). As listed in Table 2, genotypes G56, G54, and G46 had the highest SeDW values, 0.162, 0.093, and 0.148, respectively, while genotypes G69, G77, and G57 had the lowest SeDW with values of 0.090, 0.051, and 0.079, respectively, under normal and drought conditions (D1 and D2).

### Correlation among the seedling traits

**Figure 3:**
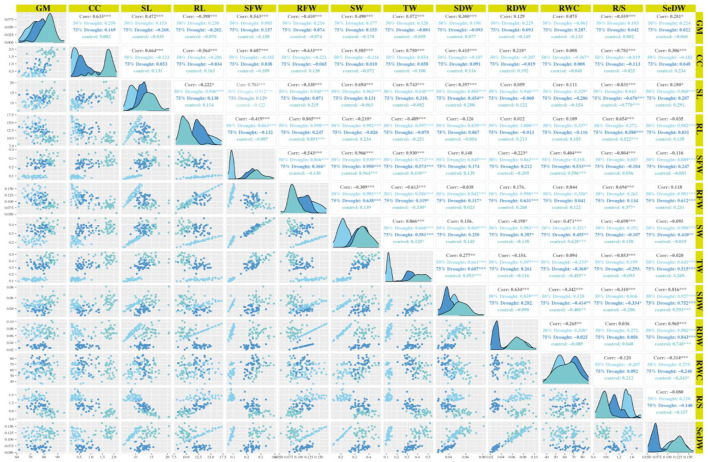
Correlations matrix of 40 genotypes at seedling stage under normal and drought conditions. GP, germination percentage; RDW, root dry weight; RFW, root fresh weight; RL, root length; RWC, relative water content; SDW, shoot dry weight; SFW, shoot fresh weight; SL, shoot length; SeDW, seedling dry weight; SDFW, seedling fresh weight; CC, chlorophyll content.

In the experiment of this study, the root fresh weight had positive and highly significant association with the root dry weight under the stress and non-stress conditions (Figure 3). However, under normal and drought conditions (D2), seedling weight was positively and highly significantly correlated with root length, whereas seedling length was correlated negatively and had significant association with root length under drought stress level D1.

The seedling weight had positive and highly significant association with shoot fresh weight under normal condition and both stressed environments as shown in Figure 3. The relative water content had positive and highly significant correlation with the seedling weight and shoot fresh weight but indicated a negative association with root fresh weight under the non-stressed condition as shown in Figure 3. Under D1 condition, the relative water content showed highly significant and a positive association with seedling weight and shoot fresh weight and exhibited a negative and significant correlation. In D2 condition, the relative water content indicated the positive and highly significant association with seedling weight, shoot fresh weight, and root fresh weight as shown in Figure 3. The root shoot ration correlated positively and showed highly significant association with root length, root fresh weight, and root dry weight under all conditions. While root shoot ratio showed a negative and highly significant association with shoot length, shoot fresh weight, and shoot dry weight under normal and D1 conditions, in D2 condition, root shoot ratio showed a positive and highly significant correlation as mentioned in Figure 3. The chlorophyll content showed highly significant and positive association with the shoot length under normal and D1 conditions and correlated negatively and significant in D2 condition (Supplementary Table S3).

### Principal component analysis

The 40 wheat genotypes of wheat were studied through principal component analysis (PCA) based on the correlation matrix to describe the diversity of the germplasm and the relationship of wheat seedling indices under normal and drought stress conditions. For parental selection in breeding programs, biplot analysis was previously used for this purpose. The first five PCs found that eigenvalues were larger than 1 mean was considered significant under normal conditions and drought level 1. The other eight (PCs) data consider as non-significant and were not useable for further analysis due to eigenvalues less than 1 under normal and D1 condition. While under drought stress D2 the first three PCs were significant and the other ten were non-significant. The first five PCs showed 82.28% of total variation under normal conditions. While under drought D1, the first five PCs showed 83.06% of total variation. Meanwhile, under drought level D2, the first two PCs showed 67.35% of total variation. The first PCs marked for 22.63% of the variance under normal conditions, 30.90% of the variance under D1 conditions, and 64.38% of the variance under D2 conditions (Table 3).

**Table 3:** Eigenvalue, variability, and cumulative of wheat seedling traits under normal and drought conditions.

	**Conditions**	**PC1**	**PC2**	**PC3**	**PC4**	**PC5**
Eigenvalue	N	2.943	2.627	2.406	1.534	1.193
	D1	4.018	2.388	2.005	1.356	1.032
	D2	7.726	1.557	1.032	0.838	0.528
Variability (%)	N	22.637	20.209	18.509	11.797	9.179
	D1	30.905	18.373	15.426	10.430	7.941
	D2	64.386	12.977	8.604	6.984	4.398
Cumulative %	N	22.637	42.846	61.355	73.152	82.331
	D1	30.905	49.278	64.704	75.133	83.074
	D2	64.386	77.362	85.966	92.950	97.349

**Figure 4:**
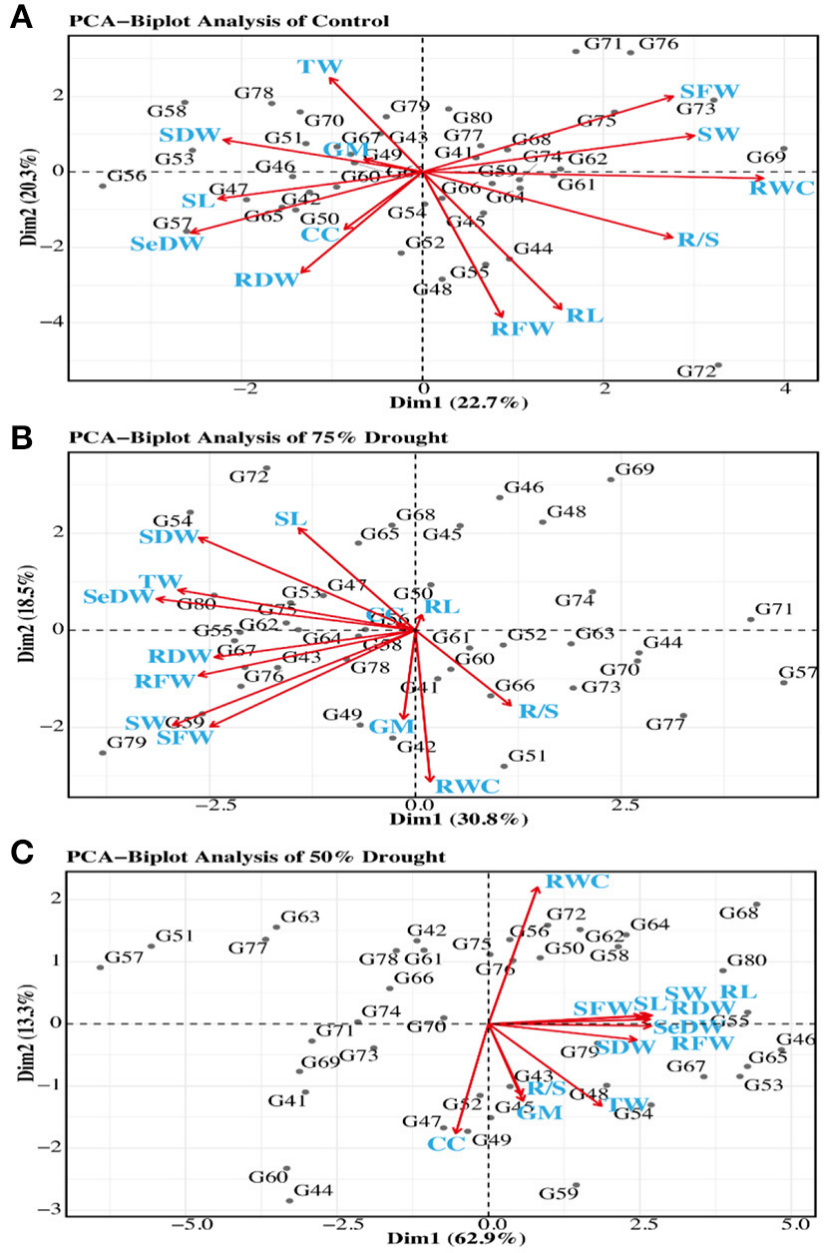
Distribution of 40 wheat genotypes on the genotype score plot PC1 and PC2 under normal **(A)**, 75% drought [**(B)**, D2], and 50% drought [**(C)**, D1] conditions.

According to the PCA which showed that the trait chlorophyll content was positively associated with seedling dry weight, root dry weight, and shoot length under normal conditions, meanwhile these traits were positively correlated with RFW, RL, RSR, RWC, SDFW, GP, and SFW. However, these all studies traits were negatively interlinked with TW and SDW. While under D2 conditions, the traits RSR was positively associated with germination percentage, chlorophyll content, shoot length, root length, shoot fresh weight, root fresh weight, seedling weight, shoot dry weight, root dry weight, turgid weight, relative water content, and seedling dry weight. However, under drought conditions (D2), the chlorophyll content is positively correlated with most of the studied traits (Figure 4). A biplot was generated between only two main factors or PCs because the first two components had maximum variability among all principal components. The biplot had four main axes, with the upper right axis having a positive impact on PC1 and PC2, and the genotypes located in that block are best for selection because the varieties in that block have the highest variation among all studied germplasm as shown in Figure 4.

## Discussions

Drought is an abiotic stress that limits plant growth and development which ultimately reduces the yield. Crops showed different physiological and morphological responses to tackle drought stress. Breeding for drought tolerance is an efficient method to overcome drought stress. Wheat breeders have developed various drought-tolerant wheat genotypes in the recent years to improve the plant performance under drought conditions (Mickky and Aldesuquy, 2017). Screening of germplasm is an excellent way to discover advance material to assist these breeding programs (Ahmad et al., 2022). In this study, 40 wheat genotypes were considered for detailed study against drought stress conditions. The increased drought stress had a significant impact on wheat germination percentage. The germination was delayed, reduced or even stopped completely due to water stress. Water is primarily required for the maintenance of turgor, which is critical for cell enlargement and growth, as well as the morphology of plants (Kramer and Boyer, 1995).

As drought stress levels increased, there was a significant difference in root and shoot fresh weight noted. Root and shoot fresh weight decreased with increasing drought stress level. Drought stress change osmotic potential of the cell, that leads to poor cell division which ultimately decrease root and shoot fresh weight (Taiz et al., 2015; Paul et al., 2018).

Relative water content is the most important parameter to know plant water status. Relative water content was significantly decreased with increasing level of osmatic stress. Many scientists reported similar result for relative water content (Ahmed et al., 2019, 2020). Researcher reported that the fall in RWC in drought-stressed conditions may be due to reduction in plant vigor (Kapoor et al., 2020).

Plant growth parameters such as shoot length and root length, as well as fresh weight, are regarded as important indices for selecting drought-tolerant wheat genotypes (Faisal et al., 2017). Under water deficit conditions, all genotypes showed a reduction in shoot and root length, seedling fresh and dry weight. Many researchers reported similar results of shoot length (Faisal et al., 2017). A little bit increasing trend was seen in root length because under stress conditions plant tend to survive and may showed increased root length.

The root/shoot ratio (RSR) showed crops relative root and shoot growth pattern. In drought stress conditions the RSR was increased significantly. A higher RSR showed that seedling root growth is less affected than seedling shoot growth under drought stress conditions (Mohi-Ud-Din et al., 2021). Under osmotic stress, the root–shoot ratio increased to improve water absorption, which is linked to abscisic acid (ABA) concentrations in roots and shoots (Nezhadahmadi et al., 2013). Wheat seedling dry weight is a significant trait that is also affected by water stress conditions. Seedling dry weight is decreased in drought stress conditions. Seedling dry matter was reduced and significantly affected by drought stress conditions (Mujtaba et al., 2016; Ahmed et al., 2019, 2020).

Chlorophyll is the primary component of photosynthesis and one of the physiological mechanisms that is most vulnerable to environmental stress (Hussain et al., 2019). In this study, the chlorophyll content was also reduced with increasing drought stress level. Drought reduces or destroys chlorophyll content, which produces reactive oxygen species (ROS) such as ${\text{O}}_{2}^{-}$ and H_2_O_2_, which can lead to lipid peroxidation (Khalilzadeh et al., 2016). Drought stress causes leakage of these electrolyte and lipid peroxidase from thylakoid membrane of chloroplast that leads to chlorophyll loss in plants (Djanaguiraman et al., 2010). The drought resistant varieties retain these pigments in their thylakoid membrane and survive in drought stress (Kalaji et al., 2016; Bala and Sikder, 2017). Reduction in chlorophyll content was also reported by the researchers (Ahmed et al., 2019; Qayyum et al., 2021).

Correlation analysis explained the relationship between two variables that useful in plant sciences because it provides links that can be used to study the relationship between many traits (Ahmed et al., 2019). The correlation between seedling attributes like root length, shoot length, root fresh weight, shoot fresh weight, root dry weight, shoot dry weight, relative water content, and chlorophyll content were studied in this experiment. Understanding the correlation among these traits was very important to improve the efficiency of breeding for drought tolerance in wheat (Sallam et al., 2019).

In this study, the root length was positively correlated with the root shoot ratio, fresh weight, and dry weight while the negative association was seen with the shoot length and relative water content. Similar result of correlation analysis was also seen by different researchers (Ahmed et al., 2019, 2020). Experiment was done by the scientists (Khan et al., 2013), they discovered a positive and highly significant association between SFW and SDW under normal and drought conditions.

Principal component (PC) analysis is a multivariate statistical analysis for examining and simplifying complex and large datasets. Only the PCs that exhibited eigenvalues higher than 1 were measured as significant. This analysis transforms the larger number of correlated variables into smaller ones, as described by Kamel et al. (2009). Jaynes et al. (2003); Ali et al. (2015) and Sisodia and Rai (2017) described that a biplot analysis can be utilized to select variables that can be categorized into main groups and subgroups based on homogeneity and dissimilarity. The PCA was based on the different principal components and each principal component has its eigenvalue which makes a percentage contribution to it (Table 3). The principal component that showed eigenvalues higher than 1 are considered significant while others are non-significant (Ahmed et al., 2019; Pour-Aboughadareh et al., 2021). The PCA results in this study was also similar to the findings reported by wheat scientists (Pour-Aboughadareh et al., 2019). The reason for a negative effect of relative water and chlorophyll content may be due to the deficiency of RWC in the cell as water deficiency in the cell induces leakage of electrolyte and peroxidase of lipids from the thylakoid membrane of chloroplast which leads to loss of chlorophyll content that has a negative impact on all other traits (Ristic et al., 2007; Djanaguiraman et al., 2010). All major traits have a negative impact on the second principal component in drought as well as normal conditions; these results are supported by earlier findings (Marček et al., 2021) in wheat crops using the seedling attributes. The germination percentage has a positive impact because as the germination increase, all other parameters are also increased (Figure 4). The major contribution for variability in PC4 under normal and D2 conditions is given by VI and SC, respectively. The principal component analysis is also helpful in selecting diverse parents for hybridization and other plant breeding techniques. The projection of genotypes on PC1 and PC2 was useful in the selection of the diverse groups of parents. The projected pattern of genotypes on the two PCs showed the population structure under normal and drought conditions (Marček et al., 2021). The studied traits are thus recognized as drought tolerance indicators for varietal selection, and varieties showing less reduction under drought could be used as a standard check in breeding programs to identify lineages with drought tolerance and could be recommended for drought-stressed areas.

## Conclusion

Drought tolerance is a highly complex trait and one of the important components of yield stability in wheat. In this study, all 40 wheat genotypes were grown using CR design under normal and drought stress levels (D1 50% field capacity and D2 75% field capacity) for the selection of drought-tolerant genotypes. The seedling attributes like root length, shoot length, root fresh weight, shoot fresh weight, root dry weight, shoot dry weight, chlorophyll content was studied under these drought stress conditions. These traits shoed positively association and selection of any one of these traits enhances the performance of other traits. Genotypes G47, G48, G65, G68, and G80 showed best performance for various traits under drought stress conditions. The principal component analysis showed that out of 13 PCs, only first five PCs showed eigenvalue greater than 1 and considered as significant except D2 which has three significant PCs. The five PCs showed 82.33, 83.07, and 97.34% cumulative percentage under control, D1, and D2 stressed conditions, respectively. These findings can be used as selection criteria for drought stress conditions to develop better drought-tolerant cultivar.

## Data availability statement

The original contributions presented in the study are included in the article/supplementary material, further inquiries can be directed to the corresponding authors.

## Author contributions

HA, YZ, AS, and MA contributed in conceptualization, project administration, methodology, data curation, formal analysis, investigation, and writing - original draft. MY, AU, and MA contributed in writing - review and editing, investigation, visualization, and formal analysis. All authors contributed to the article and approved the final version.

## Conflict of interest

The authors declare that the research was conducted in the absence of any commercial or financial relationships that could be construed as a potential conflict of interest.

## Publisher's note

All claims expressed in this article are solely those of the authors and do not necessarily represent those of their affiliated organizations, or those of the publisher, the editors and the reviewers. Any product that may be evaluated in this article, or claim that may be made by its manufacturer, is not guaranteed or endorsed by the publisher.
