# Supplementary material for: Conferring of drought tolerance in wheat (Triticum aestivum L.) genotypes using seedling indices
Source: Front Plant Sci. 2022 Jul 22;13:961049. doi: 10.3389/fpls.2022.961049 (PMC9355593; doi:10.3389/fpls.2022.961049)
Supplement: Supplementary file 1 [file Data_Sheet_1.docx]

**Table S1: Genotype Code, Name and Pedigree of studied genotypes**

| Sr. No | Code | Name | Pedigree |
| --- | --- | --- | --- |
| 1 | G41 | Watan | LU26/HD 2179 |
| 2 | G42 | AARI-2011 | SH-88/90A-204//MH97 |
| 3 | G43 | Kohinoor-83 | ORE F1 158/FDL//MFN/2*TIBA63/3/COC |
| 4 | G44 | Chakwal-86 | FORLANI/ACC//ANA or Fln/ACS//ANA |
| 5 | G45 | Uqab-2000 | CROW'S'/NAC//BOW'S' |
| 6 | G46 | Bahawal-97 | PFAU'S'/SERI |
| 7 | G47 | Chakwal-50 | ATTILA/3/HUI/CARC//CHEN/CHTO/4/ATTILA |
| 8 | G48 | AS-2002 | KHP/D31708//CMH74A370/3/ENO79/4/R26043/*4NAC |
| 9 | G49 | Bakhtawar-93 | AU/UP301//GLL/SX/3/PEW/4/MAI/MAYA//PEW |
| 10 | G50 | Pasban-90 | INIA F66/TH.DISTICHUM//INIAF66/3/GENARO T81 or INIA F66/A.DISTCHUM//INIA66/3/GEN |
| 11 | G51 | Bakhtawar-94 | Mentana/Mayo//4-11 |
| 12 | G52 | Bakhar-2002 | P102/PIMA//F371/TTR/BOW/3/PVN |
| 13 | G53 | Iqbal-2000 | BURGUS/SORT 12-13//KAL/BB/3/PAK 81 |
| 14 | G54 | Shafaq-2006 | LU 26/HD 2179/ 2*INQALAB 91 |
| 15 | G55 | Fakhar-e-Sarhad | NORD-DESPREZ(ND)/VG-9144//KALYANSONA/BLUEBIRD/3/YACO/4/VEERY-5 |
| 16 | G56 | Fareed-2006 | PT'S'/3/TOB/LFN//BB/4/BB/HD-832-5//ON/5/G-V/ALD'S'//HPO |
| 17 | G57 | FSD-2008 | PBW65/2*Pastor |
| 18 | G58 | BARS-2009 | PFAU/SERI//BOW |
| 19 | G59 | FSD-85 | MAYA/MON//KVZ/TRM |
| 20 | G60 | GA 2002 | DWL5023/SNB//SNB |
| 21 | G61 | Galaxy-2013 | Pb96/Watan/MH-97 |
| 22 | G62 | Gomal-2008 | Attila |
| 23 | G63 | Hashim-2008 | JUP/ALD'S'//KLT'S'/3/VEE'S'/6/BEZ//TOB/8156/4/ON/3/6*TH/KF//6*LEE/KF/--------- |
| 24 | G64 | Inq-91 | WL 711/CROW "S" |
| 25 | G65 | Millat-2011 | CHENAB2000/INQ-91 |
| 26 | G66 | Khyber-87 | KVZ/TRM//PTM/ANA |
| 27 | G67 | Kohistan-97 | V-1562//CHRC'S'/HORK/3/KUFRA-I/4/CARP'S'/BJY'S' |
| 28 | G68 | Aas-2011 | PRL/PASTOR//2236 |
| 29 | G69 | Kohsar-95 | PSN/BOW |
| 30 | G70 | Lasani-2008 | LUAN/KOH-97 |
| 31 | G71 | Ufaq-2002 | V.84133/V83150 |
| 32 | G72 | Marvi-2000 | CMH-77A917/PKV 1600//RL6010/6*SKA |
| 33 | G73 | Maxi-Pak 65 | PJ/GB55 |
| 34 | G74 | Mehran-89 | KVZ/BUHO//KAL/BB |
| 35 | G75 | FSD-83 | FURY//KAL/BB |
| 36 | G76 | Mairaj-08 | SPARROW/INIA//V.7394/WL711/3/BAUS |
| 37 | G77 | Moomal-2002 | BUC or BUCS/4/TZPP/IRN46 |
| 38 | G78 | Pak-81 | VEERY. |
| 39 | G79 | Parwaz-94 | V.5648/PARULA or V.5648/PRL |
| 40 | G80 | Chakwal-97 | BUC'S'/FCT'S' |

Table S2. Descriptive statistics for 40 wheat genotypes under normal and drought conditions

| Traits | Conditions | Maximum | Minimum | Mean | SD |
| --- | --- | --- | --- | --- | --- |
| GM (%) | N | 94.67 | 67.33 | 82.86 | 4.82 |
|  | D1 | 84.67 | 61.67 | 76.02 | 4.85 |
|  | D2 | 81.67 | 61.22 | 72.5 | 5.01 |
| RL (cm) | N | 14.33 | 7.87 | 9.92 | 1.2 |
|  | D1 | 15 | 8.3 | 12.49 | 1.37 |
|  | D2 | 17.2 | 8.1 | 12.94 | 2.27 |
| SL (cm) | N | 20.47 | 9.67 | 14.36 | 2.32 |
|  | D1 | 15.6 | 8.4 | 12.15 | 1.43 |
|  | D2 | 11.9 | 6.2 | 9.21 | 1.44 |
| R/S R | N | 1.12 | 0.46 | 0.72 | 0.135 |
|  | D1 | 1.75 | 0.75 | 1.08 | 0.193 |
|  | D2 | 1.88 | 1.3 | 1.48 | 0.131 |
| CC | N | 2.11 | 0.58 | 1.66 | 0.33 |
|  | D1 | 1.35 | 0.35 | 0.71 | 0.28 |
|  | D2 | 1.02 | 0.13 | 0.37 | 0.19 |
| SFW | N | 0.394 | 0.215 | 0.279 | 0.045 |
|  | D1 | 0.337 | 0.156 | 0.234 | 0.036 |
|  | D2 | 0.111 | 0.066 | 0.089 | 0.011 |
| RFW | N | 0.12 | 0.054 | 0.085 | 0.012 |
|  | D1 | 0.134 | 0.076 | 0.109 | 0.014 |
|  | D2 | 0.168 | 0.079 | 0.126 | 0.022 |
| SDFW | N | 0.469 | 0.295 | 0.365 | 0.045 |
|  | D1 | 0.47 | 0.26 | 0.344 | 0.044 |
|  | D2 | 0.272 | 0.146 | 0.216 | 0.032 |
| SDW | N | 0.081 | 0.031 | 0.054 | 0.01 |
|  | D1 | 0.047 | 0.027 | 0.037 | 0.01 |
|  | D2 | 0.064 | 0.035 | 0.046 | 0.01 |
| RDW | N | 0.103 | 0.052 | 0.071 | 0.012 |
|  | D1 | 0.052 | 0.02 | 0.031 | 0.01 |
|  | D2 | 0.094 | 0.044 | 0.071 | 0.013 |
| RWC | N | 84.44 | 39.5 | 61.73 | 11.1 |
|  | D1 | 86.38 | 43.96 | 67.87 | 10.52 |
|  | D2 | 73.42 | 35.71 | 57.57 | 9.85 |
| SDDW | N | 0.162 | 0.09 | 0.125 | 0.014 |
|  | D1 | 0.093 | 0.051 | 0.068 | 0.01 |
|  | D2 | 0.148 | 0.079 | 0.117 | 0.018 |

**GP** = germination percentage, **CC** = Chlorophyll Content, **SL**=shoot length, **RL**=root length, **SFW**= shoot fresh weight, **RFW** = root fresh weight, **SDFW** = seedling fresh weight, **SDDW** = shoot dry weight, **RDW** = root dry weight, **RWC**= relative water content, **R/S R** = Root shoot ratio, **SDDW** = Seedling dry weight.

**Table S3: Correlations Matrix of 40 genotypes under seedling traits Under Normal and Drought Conditions**

| **Traits** | **Level** | **GP** | **CC** | **SL** | **RL** | **SFW** | **RFW** | **SeW** | **SDW** | **RDW** | **RWC** | **RSR** |
| --- | --- | --- | --- | --- | --- | --- | --- | --- | --- | --- | --- | --- |
| **CC** | Normal | 0.0017 |  |  |  |  |  |  |  |  |  |  |
|  | Drought 1 | 0.1707 |  |  |  |  |  |  |  |  |  |  |
|  | Drought 2 | 0.2582 |  |  |  |  |  |  |  |  |  |  |
| **SL** | Normal | -0.0352 | 0.135** |  |  |  |  |  |  |  |  |  |
|  | Drought 1 | -0.2641^**^ | 0.052** |  |  |  |  |  |  |  |  |  |
|  | Drought 2 | 0.152 | -0.1236* |  |  |  |  |  |  |  |  |  |
| **RL** | Normal | -0.0693 | 0.1637 | 0.1128 |  |  |  |  |  |  |  |  |
|  | Drought 1 | -0.2807^**^ | -0.0313 | 0.1301 |  |  |  |  |  |  |  |  |
|  | Drought 2 | 0.2288 | -0.2098 | 0.9443^**^ |  |  |  |  |  |  |  |  |
| **SFW** | Normal | -0.1498 | -0.1093 | -0.1203 | -0.0066 |  |  |  |  |  |  |  |
|  | Drought 1 | 0.1584 | 0.0337 | 0.1266 | -0.1346 |  |  |  |  |  |  |  |
|  | Drought 2 | 0.0832 | -0.1896 | 0.9115^**^ | 0.8607^**^ |  |  |  |  |  |  |  |
| **RFW** | Normal | -0.0788 | 0.1388 | 0.2218 | 0.8887^**^ | -0.1319 |  |  |  |  |  |  |
|  | Drought 1 | 0.0694 | -0.0628 | 0.0671 | 0.2418 | 0.3676^**^ |  |  |  |  |  |  |
|  | Drought 2 | 0.2154 | -0.2206 | 0.9442^**^ | 0.9975^**^ | 0.8637^**^ |  |  |  |  |  |  |
| **SeW** | Normal | -0.1709 | -0.0715 | -0.0608 | 0.2333** | 0.9633^**^ | 0.1388 |  |  |  |  |  |
|  | Drought 1 | 0.1552 | 0.0076 | 0.1279 | -0.0276* | 0.9499^**^ | 0.6397^**^ |  |  |  |  |  |
|  | Drought 2 | 0.1751 | -0.218 | 0.9632^**^ | 0.9818^**^ | 0.9387^**^ | 0.9844^**^ |  |  |  |  |  |
| **SDW** | Normal | 0.0778 | 0.1245 | 0.2774^**^ | -0.0463 | 0.1364 | 0.0373 | 0.1465 |  |  |  |  |
|  | Drought 1 | -0.0986 | 0.0961 | 0.4479^**^ | 0.0671 | 0.1921 | 0.3147^**^ | 0.2643 |  |  |  |  |
|  | Drought 2 | 0.1778 | -0.1169 | 0.879^**^ | 0.8324^**^ | 0.8396^**^ | 0.835^**^ | 0.8639^**^ |  |  |  |  |
| **RDW** | Normal | -0.1424 | 0.1986 | 0.1166 | 0.2052 | -0.2102 | 0.2504** | -0.1419 | -0.0828 |  |  |  |
|  | Drought 1 | 0.0889 | -0.0256 | -0.0707 | -0.01 | 0.2092 | 0.632^**^ | 0.3836^**^ | 0.282^**^ |  |  |  |
|  | Drought 2 | 0.2346 | -0.2124 | 0.9429^**^ | 0.9996^**^ | 0.8591^**^ | 0.9976^**^ | 0.9813^**^ | 0.8342^**^ |  |  |  |
| **RWC** | Normal | -0.1331 | -0.0387 | -0.1537 | 0.185 | 0.595^**^ | 0.1147 | 0.6275^**^ | -0.4041^**^ | -0.0901** |  |  |
|  | Drought 1 | 0.2867^**^ | 0.0061 | -.02049 | -0.115 | 0.5345^**^ | 0.0395 | 0.4544^**^ | -0.4059^**^ | -0.03324* |  |  |
|  | Drought 2 | -0.0622 | -0.3673^**^ | 0.3376^**^ | 0.3371^**^ | 0.3139^**^ | 0.3544^**^ | 0.3509^**^ | 0.1132 | 0.336^**^ |  |  |
| **RSR** | Normal | 0.0057 | -0.0326 | -0.7695^**^ | 0.5227 | 0.0493 | 0.3729^**^ | 0.1503 | -0.2732^**^ | 0.0414 | 0.2068 |  |
|  | Drought 1 | 0.0375 | -0.1109 | -0.6755^**^ | 0.586^**^ | -0.1843 | 0.1307 | -0.1071 | -0.3351^**^ | 0.0645 | 0.0929 |  |
|  | Drought 2 | 0.1862 | -0.0178 | 0.0449 | 0.2779 | 0.0399 | 0.2705^**^ | 0.1979 | 0.07 | 0.2813^**^ | -0.2072 |  |
| **SeDW** | Normal | -0.0722 | 0.2354 | 0.2938^**^ | 0.1371 | -0.0832 | 0.2341 | -0.0195 | 0.6031^**^ | 0.7441^**^ | -0.345^**^ | -0.1599 |
|  | Drought 1 | 0.0264 | 0.0479 | 0.2059 | 0.0307 | 0.2473 | 0.614^**^ | 0.4096^**^ | 0.749^**^ | 0.8436^**^ | -0.2346 | -0.1393 |
|  | Drought 2 | 0.2228 | -0.1797 | 0.9601^**^ | 0.9815^**^ | 0.8889^**^ | 0.9809^**^ | 0.9804^**^ | 0.922^**^ | 0.982^**^ | 0.2714^**^ | 0.2155 |

GP=Germination Percentage, RDW=Root dry weight, RFW=Root fresh weight, RL=Root Length, RWC= Relative water content, SDW=Shoot dry Weight, SFW=Shoot fresh weight, SL=shoot length, SeDW=Seedling dry weight, SeW=Seedling Weight, CC=Chlorophyll Content
